# Reduced Expression of *Slc* Genes in the VTA and NAcc of Male Mice with Positive Fighting Experience

**DOI:** 10.3390/genes12071099

**Published:** 2021-07-20

**Authors:** Dmitry A. Smagin, Vladimir N. Babenko, Olga E. Redina, Irina L. Kovalenko, Anna G. Galyamina, Natalia N. Kudryavtseva

**Affiliations:** 1 Neuropathology Modeling Laboratory, The FRC Institute of Cytology and Genetics SB RAS, 630090 Novosibirsk, Russia; smagin@bionet.nsc.ru (D.A.S.); bob@bionet.nsc.ru (V.N.B.); oredina@bionet.nsc.ru (O.E.R.); koir@bionet.nsc.ru (I.L.K.); galyamina@bionet.nsc.ru (A.G.G.); 2 Neurogenetics of Social Behavior Sector, The FRC Institute of Cytology and Genetics SB RAS, 630090 Novosibirsk, Russia

**Keywords:** ventral tegmental area, nucleus accumbens, prefrontal cortex, *Slc* gene family, repeated aggression, positive fighting experience, gene expression, RNA-Seq

## Abstract

A range of several psychiatric medications targeting the activity of solute carrier (SLC) transporters have proved effective for treatment. Therefore, further research is needed to elucidate the expression profiles of the *Slc* genes, which may serve as markers of altered brain metabolic processes and neurotransmitter activities in psychoneurological disorders. We studied the *Slc* differentially expressed genes (DEGs) using transcriptomic profiles in the ventral tegmental area (VTA), nucleus accumbens (NAcc), and prefrontal cortex (PFC) of control and aggressive male mice with psychosis-like behavior induced by repeated experience of aggression accompanied with wins in daily agonistic interactions. The majority of the *Slc* DEGs were shown to have brain region-specific expression profiles. Most of these genes in the VTA and NAcc (12 of 17 and 25 of 26, respectively) were downregulated, which was not the case in the PFC (6 and 5, up- and downregulated, respectively). In the VTA and NAcc, altered expression was observed for the genes encoding the transporters of neurotransmitters as well as inorganic and organic ions, amino acids, metals, glucose, etc. This indicates an alteration in transport functions for many substrates, which can lead to the downregulation or even disruption of cellular and neurotransmitter processes in the VTA and NAcc, which are attributable to chronic stimulation of the reward systems induced by positive fighting experience. There is not a single *Slc* DEG common to all three brain regions. Our findings show that in male mice with repeated experience of aggression, altered activity of neurotransmitter systems leads to a restructuring of metabolic and neurotransmitter processes in a way specific for each brain region. We assume that the scoring of *Slc* DEGs by the largest instances of significant expression co-variation with other genes may outline a candidate for new prognostic drug targets. Thus, we propose that the *Slc* genes set may be treated as a sensitive genes marker scaffold in brain RNA-Seq studies.

## 1. Introduction

The solute carrier (SLC) group of membrane transport proteins comprises over 400 human disease-associated genes organized into 65 families [1,2,3,4]. SLCs are responsible for transporting extremely diverse solutes, including neurotransmitters, organic molecules as well as inorganic ions, metals, etc. Most SLC transporters are located in the cell membrane, and some of them are located in the mitochondria or in other intracellular organelles.

Several SLC transporters are targets for well-known drugs [3,5] for the treatment of psychoneurological disorders. As an example, inhibitors of SLC6A transporters (SLC6A2, SLC6A3, and SLC6A4 proteins) are promising agents for depression treatment, which act by reducing monoamines uptake in the synapses, thereby increasing their levels in the synaptic cleft. Inhibitors of the vesicular monoamine SLC18a2 carrier, which transports monoamines into synaptic vesicles, is the treatment option for Huntington’s disease [6] and psychostimulant abuse and addiction [7]. The inhibition of SLC6A9 and SLC6A5 transporters, which regulate extracellular glycine levels in brain tissue by neuronal inhibition and excitation, is used for reducing schizophrenia symptoms, which are hypothesized to be due to a deficiency in glutamatergic signaling, since glycine also binds to excitatory NMDA receptors [8]. Therefore, the altered expression of *Slc* genes may be indicative of altered metabolic processes and neurotransmitter systems activity in patients at risk of developing psychiatric disorders.

Our previous studies have shown that male mice winning in repeated daily agonistic interactions develop a psychosis-like state, exhibiting symptoms of pathological behaviors: abnormal aggression in any social situation, irritability, high impulsivity, disturbances of social recognition, enhanced anxiety and other symptoms, such as repeated stereotypies, hyperactivity, etc. [9,10,11,12,13,14]. Altered metabolism and neurotransmitter systems activity, as well as changes in the expression of several key genes, were found in several brain regions of chronically aggressive mice [15,16,17,18,19]. Increased expression of dopaminergic genes (*Th*, *Slc6a3*, *Snca*) against the background of activation of brain dopaminergic systems [20], was found in the ventral tegmental area [15,17], which contains the bodies of dopaminergic neurons. The differentially expressed genes (DEGs) encoding proteins involved in the metabolism of the GABAergic and glutamatergic systems were found in the dorsal striatum [13]. It is well known that these brain regions are responsible for the mechanisms of positive reinforcement, in particular, accompanying aggressive behavior.

The goal of this study is to analyze the expression of *Slc* genes and to identify those with changed expression in brain regions of male mice under repeated experience of aggression accompanied by wins: ventral tegmental area (VTA), ventral striatum (nucleus accumbens: NAcc), and prefrontal cortex (PFC), i.e., the brain regions involved in sex, food, and drug reward systems as well as in aggression and addiction [21,22,23,24,25,26,27]. We assume that *Slc* DEGs can serve as the markers of altered functions in the brain regions and as possible treatment targets for psychiatric diseases, in particular, psychosis accompanied by aggression.

## 2. Materials and Methods

Adult male mice C57BL/6 were obtained from the Animal Breeding Facility, Branch of the Institute of Bioorganic Chemistry of the RAS (Pushchino, Moscow region). Animals were housed under standard conditions (12:12 h light/dark regime starting at 8:00 a.m., at a constant temperature of 22 ± 2 °C, with food in pellets and water available ad libitum). Mice were weaned at three weeks of age and housed in groups of 8–10 in standard plastic cages (36 cm × 23 cm × 12 cm). Experiments were performed with 10–12 week old animals. All procedures were in compliance with the European Communities Council Directive 210/63/EU on 22 September 2010. The study was approved by Scientific Council N 9 of the Institute of Cytology and Genetics SB RAS of 24 March 2010, N 613 (Novosibirsk).

### 2.1. Generation of Repeated Aggression in Male Mice

Repeated negative and positive social experiences in male mice were induced by daily agonistic interactions with use of sensory contact model, which was later renamed as the “model of chronic social conflicts” [11,28]. Pairs of male mice were each placed in a cage (28 cm × 14 cm × 10 cm) bisected by a perforated transparent partition allowing the animals to hear, see, and smell each other, but preventing physical contact. The animals were left undisturbed for two days to adapt to new housing conditions and sensory acquaintance before they were exposed to agonistic interactions. Every afternoon (2:00–5:00 p.m. local time), the cage cover was replaced by a transparent one, and 5 min later (the time it takes for mice to start reacting to a partner in a neighboring compartment), the partition was removed for 10 min to encourage agonistic interactions. The superiority of one of the mice was firmly established within two or three confrontations with the same opponent. The superior mouse (winner) would be attacking, chasing, and biting the other, who would be displaying only defensive behavior (withdrawal, sideways postures, upright postures, freezing, or lying on the back). As a rule, aggressive interactions between males were discontinued by the lowering of the partition if the strong attacks lasted 3 min (in some cases less) to prevent the damage of the defeated mice. Each defeated mouse (loser) was exposed to the same winner for three days, while afterwards each the loser was placed, once a day after the agonistic interactions, in an unfamiliar cage with a strange winner behind the partition. Each winning mouse (aggressive mice, winner) remained in its original cage. Handling was present similarly for all groups of animals, when the litter bedding was changed in the cages every 5–6 days. This procedure was performed for 20 days (once a day) and yielded an equal number of the losers and winners. 

In each experiment, we tracked the behavior of all males, making videos of behavior during agonistic interactions, which allowed us to identify the most aggressive mice that demonstrated the daily greatest number and duration of attacks, etc. Winners with the most expressed aggressive phenotypes (long lasting expressed aggression toward any losers every day) were selected for the transcriptome analysis.

Two groups of animals were analyzed in this experiment: (1) controls—mice without a consecutive experience of agonistic interactions; (2) winners—groups of repeatedly aggressive mice. The winners, 24 h after the last agonistic interaction, and the control animals were decapitated simultaneously. The brain regions (VTA, NAcc, and PFC) were dissected by the same experimenter according to the map presented in the Allen Mouse Brain Atlas [29]. All samples were placed in RNAlater solution (Life Technologies, Waltham, MA, USA) and were stored at −70 °C until sequencing.

### 2.2. Brain Regions

Transcriptomic analysis was performed in the VTA, NAcc, and PFC of male mice. The VTA contains 55–65% of the dopaminergic cell bodies [30,31,32] giving rise to the dopaminergic mesolimbic and mesocortical pathways which project to the NAcc and PFC, respectively: dopamine is a major neurotransmitter that is involved in the integration of afferent signals with inhibitory or excitatory inputs [30,33,34]. It is suggested that the VTA could act as a hub converging and integrating multimodal signals toward dopaminergic systems [35]. GABAergic and glutamatergic neurons are also present in the VTA [36,37]. Most of the neurons in the NAcc are GABAergic medium spiny neurons (MSNs) which express D1-type or D2-type receptors [38,39]; about 1–2% are cholinergic interneurons and another 1–2% are GABAergic interneurons. GABA is the predominant neurotransmitter in the NAcc, and GABA receptors are numerous [40]. These neurons play an important role in processing reward stimuli [41]. The PFC is highly interconnected with other brain regions including through extensive connections with subcortical and other cortical structures [42]. Several neurotransmitter systems are represented in the PFC, in particular dopaminergic, glutamatergic, and cholinergic systems [43,44].

### 2.3. RNA-Seq Analysis

The collected samples were sent to JSC Genoanalytica (https://genoanalytica.ru/ accessed on 21 November 2017, Moscow, Russia). The mRNA was extracted using a Dynabeads mRNA Purification Kit (Ambion, Thermo Fisher Scientific, Waltham, MA, USA). cDNA libraries were constructed using the NEBNext mRNA Library PrepReagent Set for Illumina (New England Biolabs, Ipswich, MA USA) following the manufacturer’s protocol and were subjected to Illumina sequencing. The resulting “fastq” format files were used to align all reads to the GRCm38.p3 reference genome using the TopHat aligner [45]. The Cuffdiff suite of Cufflinks package was used to elucidate differentially expressed genes: https://www.genepattern.org/modules/docs/Cuffdiff/7, accessed on 11 July 2014. The Cufflinks program was used to estimate the gene expression levels in FPKM units (fragments per kilobase of transcript per million mapped reads) and subsequently identify the differentially expressed genes in the brain regions of male mice from affected and control groups. Each brain area of each mouse was analyzed separately in groups of 3 vs. 3 animals. Only annotated gene sequences were used in the following analysis. The level of significance at *p* < 0.05 and *q* < 0.05 were taken into consideration to define genes as differentially expressed. 

We have previously conducted studies of gene expression in males in similar experiments using the RT-PCR method with a larger number of samples for each compared experimental group, i.e., winners and losers (>10 animals). The direction and extent of changes in the experimental groups *vs*. control in the expression of the *Tph2*, *Slc6a4, Bdnf*, *Creb1*, and *Gapdh* genes in the midbrain raphe nuclei of males as determined by the two methods, RT-PCR [19,46] and RNA-Seq [47], are generally consistent. In the VTA, both methods showed similar changes in the level of transcription of genes *Тh*, *Slc6а3*, and *Snca* [17]. In order to cross-validate the results obtained, we also employed a unique resource from Stanford University, USA [48] and found a significant concordance with our RNA-Seq data pool [49]. These findings suggest that the transcriptome analyses of the data provided by JSC Genoanalytica (http://genoanalytica.ru, Moscow, Russia) have been verified, and that the method reflects the actual processes that occur in the brain under our experimental paradigm. 

### 2.4. Cell-Specific DEG Distribution

The cellular specificity of *Slc* DEGs was assessed based on the Glia specification patterns of gene expression in different brain cells: neurons, glial cells (astrocytes, oligodendrocyte precursor, newly formed oligodendrocyte, myelinating oligodendrocyte, microglia), and endothelial cells from the Barres Laboratory described in [48]. To assess the degree of the cellular specificity of DEGs, we used the values of gene expression (in FPKM). It was considered that genes predominantly affect the function of glial cells if the total expression in all types of glial cells exceeds 50% of the total expression in all types of cells. 

### 2.5. Statistical Analysis

Agglomerative hierarchical clustering (AHC) was performed using XLStat Version 2016.02 software (www.xlstat.com, accessed on 31 March 2016). The Pearson correlation coefficient has been used as a similarity metric for AHC analysis. The agglomeration method was unweighted pair-group average. Principal component analysis (PCA) was based on the Pearson correlation metric calculated on FPKM value profiles of 48 DEGs across the samples used. RNA-Seq FPKM data were used for PCA biplot analysis to assess the covariance of gene expression profiles.

## 3. Results

### 3.1. Ventral Tegmental Area

**Figure 1:**
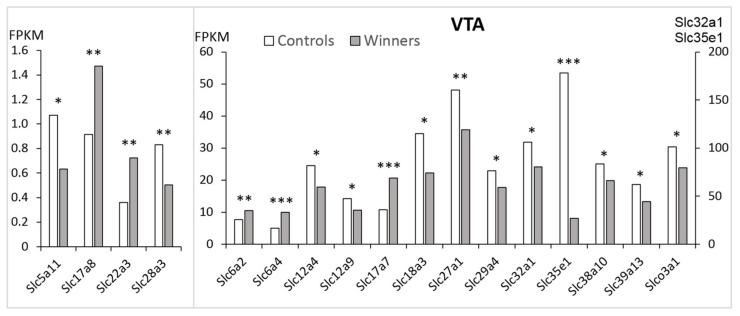
The *Slc* DEGs in the VTA of mice. White columns: controls; grey columns: winners. *: *p* < 0.05; **: *p* < 0.01; ***: *p* < 0.001. Additional information is shown in Supplementary Table S1.

In the VTA of the winners, decreased expression compared to the controls was found for twelve *Slc* DEGs: *Slc5a11, Slc12a4, Slc12a9, Slc18a3, Slc27a1, Slc28a3, Slc29a4, Slc32a1, Slc35e1, Slc38a10, Slc39a13*, and *Slco3a1* genes (Figure 1).

Genes ***Slc6a2****, **Slc6a4**, **Slc17a7**, **Slc17a8***, and ***Slc22a3*** were upregulated (hereinafter bold indicates upregulated genes). In the winners, as compared to the control mice, the most statistically significant changes were found in the expression of the *Slc6a4, Slc17a7,* and *Slc35e1* genes.

**Figure 2:**
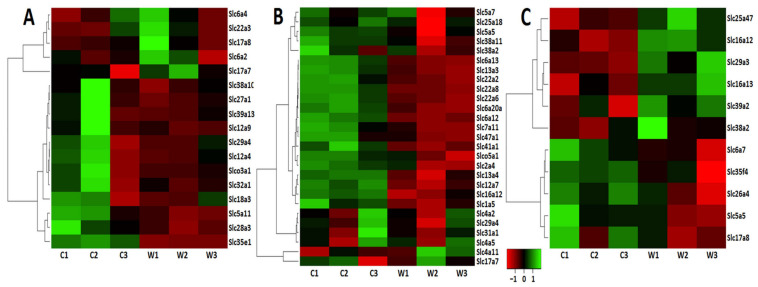
Heatmap visualization. The heatmap analysis based on expression profiles of the Slc* DEGs in the brain regions of the winners (W1, W2, W3) and control male mice (C1, C2, C3) in the VTA (**A**), NAcc (**B**), and PFC (**C**). The genes were clustered using linkage hierarchical clustering by Euclidean distance. The gene expression levels are shown with red for low, black for middle, and green for high expression levels.

Differential expression of *Slc* genes indicates transport impairments for many categories of substrates in the VTA of winning mice (Table 1). The VTA *Slc* DEGs were clustered using AHC analysis. Figure 2A presents the heatmap analysis based on expression profiles of the *Slc* DEGs. 

Three distinct clusters have been identified. AHC showed that based on highly coordinated gene expression profiles (Supplementary Table S2, VTA) the upregulated genes constitute separate clusters. One of them includes four genes associated with the regulation of neurotransmitter transport: the ***Slc6a2*** and ***Slc6a4*** genes encoding the noradrenaline and serotonin transporters, which carry out the reuptake of monoamines into the presynaptic terminals; the ***Slc17a8*** gene encoding the protein belonging to vesicular glutamate transporter family, which mediates the uptake of glutamate into synaptic vesicles at presynaptic nerve terminals of excitatory neural cells; and the ***Slc22a3*** gene encoding the protein belonging to organic cation/anion/zwitterion transporter family, which participates in dopamine transport. The second cluster was represented by a single upregulated ***Slc17a7*** gene encoding vesicular glutamate transporter, which demonstrates low association with other transport signaling. Additionally, the upregulation of the ***Slc6a3*** gene encoding dopamine transporter was shown in the VTA in previous experiments with use of RT-PCR methods [15,17]. All 12 downregulated genes were included in a separate large cluster (Figure 2A, Supplementary Table S2).

These data indicate a deficiency in the transmembrane transport of amino acids (*Slc38a10*), inorganic (*Slc12a4*, *Slc12a9*), organic and oligopeptide transporters (*Slco3a1*), glucose and nucleotide-sugar transporter (*Slc5a11*, *Slc35e1*, respectively), fatty acids (*Slc27a1*), zinc (*Slc39a13*), the nucleoside transporters which catalyze the reuptake of substrates into presynaptic neurons (*Slc28a3*, *Slc29a4*) thereby regulating the intensity and duration of monoamine neural signaling, including serotonin, dopamine, vesicular transporters, which are involved in acetylcholine and monoamine transport (*Slc18a3*), GABA and glycine (*Slc32a1*) in the VTA. 

Thus, the AHC analysis demonstrated that each cluster corresponds to a putative specific modification of the signal transduction cascade, and a significant number of analyzed DEGs are involved in the regulation of neurotransmitter events. The largest number of positive correlations in the VTA were discovered for the expression of the *Slc12a4*, *Slc29a4*, and *Slco3a1* genes (Supplementary Table S3), which may indicate the involvement of encoded proteins in all relevant coordinated processes.

### 3.2. Nucleus Accumbens

**Figure 3:**
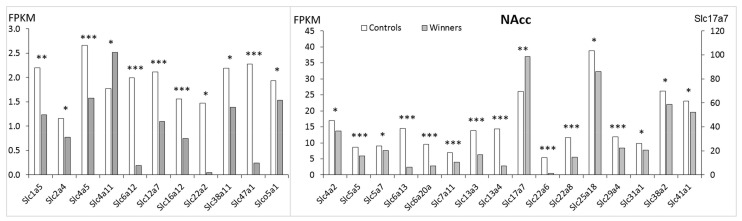
The *Slc* DEGs in the NAcc of mice. White columns: controls, grey columns: winners. *: p < 0.05; **: *p* < 0.01; ***: p < 0.001. Additional information is shown in Supplementary Table S1.

In the NAcc (Figure 3, Table S1) 27 *Slc* DEGs were identified. Only 2 genes (***Slc4a11*** and ***Slc17a7***) were upregulated, and the remaining 25 genes were downregulated. The heatmap analysis based on expression profiles of the *Slc* DEGs that were found in the winners in comparison with control mice is presented in Figure 2B. 

In the NAcc, downregulation of most DEGs indicates disturbances in transport for many substrate categories (Table 1). AHC analysis showed that downregulated genes were assigned to three separate clusters and two upregulated genes were assigned to two different clusters (Table S2, NAcc). The largest cluster was comprised of 16 genes. The genes in this cluster encode the carriers of neurotransmitter GABA (*Slc6a12*, *Slc6a13*, *Slc6a20a*), amino acids (*Slc1a5*, *Slc7a11*), transporters of inorganic ions (*Slc12a7*, *Slc13a3*, *Slc13a4*, *Slc16a12*, *Slc41a1*) and organic anions and cations (*Slc22a2*, *Slc22a6*, *Slc22a8*, *Slco5a1*), glucose transporters (*Slc2a4*) and multisubstrates, such as glucose, sugar, bile salts, organic acids, metal ions and amine compounds (*Slc47a1*). In the second cluster the downregulated genes encode inorganic ions (*Slc4a2*, *Slc4a5*), nucleoside (*Slc29a4*), and copper (*Slc31a1*) transporters. The third cluster includes amino acids transporters (*Slc38a2*, *Slc38a11*), the *Slc5a5* and *Slc5a7* genes encoding sodium iodide and choline transporters, and the mitochondrial *Slc25a18* gene.

The fourth and fifth clusters each contain one upregulated gene: ***Slc4a11*** gene, which encodes a sodium bicarbonate transporter-like protein, and ***Slc17a7*** gene, which is involved in glutamate signaling, respectively. 

In the NAcc the largest number of positive correlations with expression of other genes were found (Table S3) for the *Slc6a12* and *Slc6a13* genes encoding GABA transporters and the *Slc13a3* and *Slc22a8* genes encoding sodium-dependent decarboxylase and organic anion transporters, respectively, as well as the *Slc38a11* (putative sodium-coupled neutral amino acid transporter), which may indicate that these processes are deeply intertwined. 

Since the regulation of gene expression in the NAcc, including the studied *Slc* transporters, can be regulated by neurotransmitter signals from other regions of the brain, we evaluated the changes in the transcription of DEGs encoding neurotransmitter receptors. Differences were found in the level of transcription of six genes (Table S4). AHC analysis demonstrated that the expression of each of the genes encoding neurotransmitter receptors corresponded to a specific cluster of the *Slc* DEGs. The data obtained suggest a coordinated functioning of certain SLC transporters with the corresponding neurotransmitters.

### 3.3. Prefrontal Cortex

**Figure 4:**
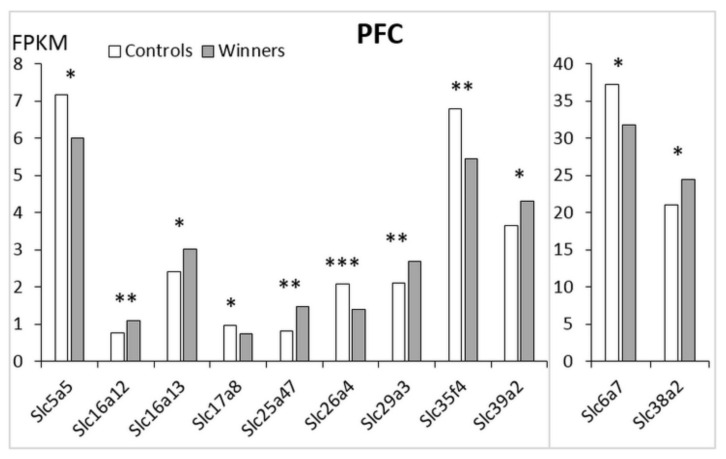
The *Slc* DEGs in the PFC of mice. White columns: controls; grey columns: winners. *: *p* < 0.05; **: *p* < 0.01; ***: *p* < 0.001. Additional information are shown in Supplementary Table S1.

In the PFC (Figure 4; Table S1) there are 11 *Slc* DEGs. Five of these genes were downregulated (*Slc5a5, Slc6a7, Slc17a8, Slc26a4, Slc35f4*) and six genes (***Slc16a12****, **Slc16a13**, **Slc25a47**, **Slc29a3**, **Slc38a2**, **Slc39a2***) were upregulated (Table 1). 

The heatmap analysis based on expression profiles of the *Slc* DEGs in the winners in the PFC is shown in Figure 2C. The AHC grouped the *Slc* DEGs into three clusters (Table S2). The first cluster combined all genes with reduced PFC expression. These genes encode transporters of sodium iodide and proline transporters (*Slc5a5* and *Slc6a7*); inorganic ions (*Slc26a4*), a putative nucleotide sugar (*Slc35f4*), and vesicular glutamate (*Slc17a8*). The second cluster includes upregulated genes encoding transporters of monocarboxylate (***Slc16a12***, ***Slc16a13***), mitochondrial and facilitative nucleoside transporters (***Slc25a47*** and ***Slc29a3***, respectively), and one that transports zinc to the cell (***Slc39a2***). The third cluster is represented by a single upregulated ***Slc38a2*** gene encoding amino acid transporters.

The upregulated ***Slc16a13*** gene showed the largest number of correlative connections, including positive correlation with the upregulated ***Slc29a3*** gene and negative correlation with all five downregulated genes: *Slc5a5*, *Slc6a7*, *Slc17a8*, *Slc26a4*, and *Slc35f4* (Table S3).

### 3.4. Overlapping of the Slc DEGs in Three Brain Structures

From the data presented in Table 1, it can be seen that there is not a single *Slc* DEG common to all three brain regions. Only two genes (*Slc29a4* and *Slc17a7*) altered their expression in the same way in both the VTA and NAcc regions. The *Slc17a8* gene was upregulated in the VTA and downregulated in the PFC. Similarly, the *Slc38a2* and *Slc16a12* gene expression was downregulated in the NAcc and upregulated in the PFC. The *Slc5a5* was downregulated in the NAcc and PFC. The other *Slc* DEGs changed level of transcription in only one of the three brain regions studied.

### 3.5. PCA of the Slc DEGs

**Figure 5:**
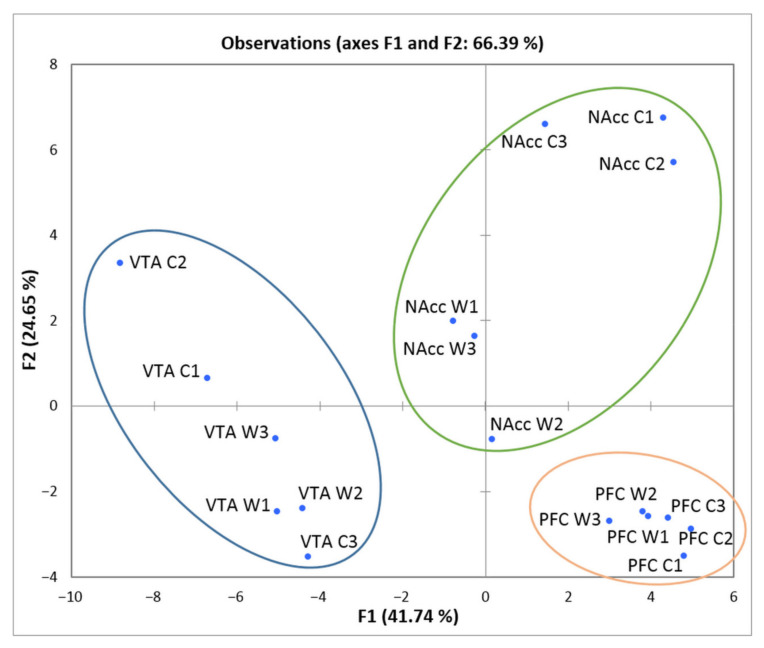
PCA plot based on covariation of genes using the expression profiles of 48 *Slc* genes across 18 samples, which comprised RNA-Seq FPKM data for three brain regions. Ovals correspond to brain regions. W1, W2, W3: winners; C1, C2, C3: controls; VTA: ventral tegmental area, NAcc: nucleus accumbens; PFC: prefrontal cortex. The figure shows a distinct clustering of the three brain regions.

To assess the degree of brain region-specific expression of genes of interest, we performed the PCA based on the covariation of 49 genes using the expression profiles of 18 samples, which comprised RNA-Seq FPKM data for three brain regions (Figure 5). 

We observed compact clustering of samples in the VTA, NAcc, and PFC based on gene expression profiles. Within the circled area one may observe the compact clustering of aggressive vs. control males, especially in the NAcc, where the vast majority of DEGs concordantly changed expression downwards in the winners. Compact clustering of samples underscores the distinct expression patterns of considered genes in each brain region.

**Figure 6:**
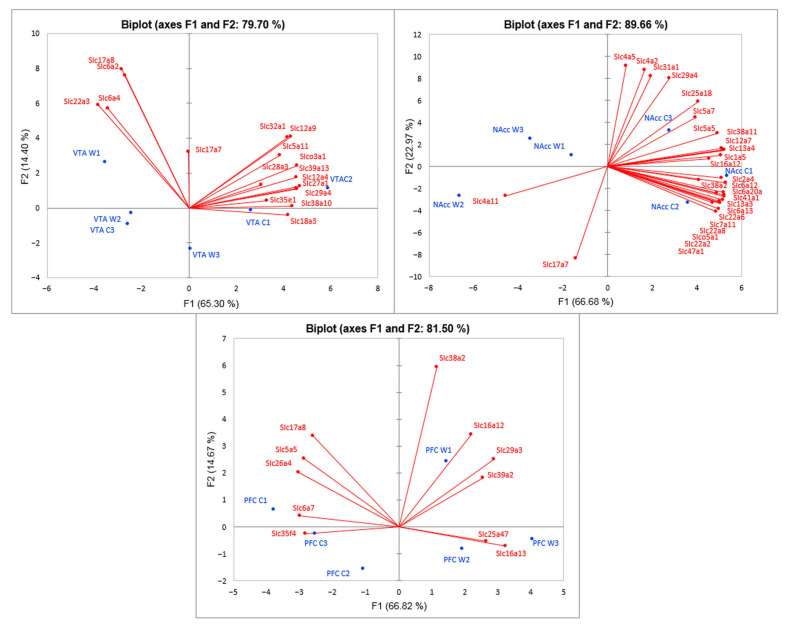
PCA biplot based on covariation of *Slc* DEGs using the expression profiles, which comprised RNA-Seq FPKM data for three brain regions. Red points: active variables; Blue points: active observations. W1, W2, W3: winners; C1, C2, C3: controls. Figure demonstrates distinct intergroup clustering of *Slc* DEGs. VTA: ventral tegmental area, NAcc: nucleus accumbens; PFC: prefrontal cortex.

The PCA biplot analysis based on covariation of the gene expression profiles of six samples for each brain region (Figure 6) demonstrated distinct intergroup clustering of *Slc* DEGs. All three graphs represent the correlated clusters of *Slc* DEGs directed oppositely, which reflects the increased and decreased levels of gene transcription.

### 3.6. Cell-Specific DEGs Distribution 

Analysis of cell-specific DEGs distribution based on Glia specification from Barres Laboratory data [48] have demonstrated (Table S5) that in the VTA and NAcc most of the *Slc* DEGs may participate in glial processes. The most significant changes in the level of transcription of *Slc* genes were found in NAcc. Here we would like to emphasize that these changes concern primarily the function of glial cells. In PFC, an increase in the level of *Slc* DEGs transcription is also associated with processes in glial cells, while a decrease in *Slc* DEGs expression is mainly associated with processes in neurons.

## 4. Discussion

It is suggested that the altered expression of the *Slc* genes encoding the transporters can serve as a marker for altered function of substrates, including neurotransmitters [3,5]. Our data confirmed the hypothesis that the expression level of genes encoding transporter proteins can be a measure of the activity of corresponding transmitters [50]. We revealed the positive correlation between the expression of genes encoding monoamine synthesis enzymes and the expression of the *Slc6a* genes encoding the corresponding transporters in brain regions. For example: the expression of the *Slc6a4* gene, encoding the serotonin transporter, significantly correlates with the expression of the *Tph2* gene, encoding the rate-limiting enzyme for serotonin synthesis. Expression of the *Slc6a2* gene, encoding the noradrenaline transporter, correlates with the expression of the *Dbh* gene encoding dopamine β-enzyme involved in the synthesis of noradrenaline from dopamine; the expression of the *Slc6a3* gene encoding the dopamine transporter was correlated with the expression of the *Th* gene, encoding the rate-limiting enzyme for dopamine synthesis. In addition, *Tph2* gene expression indices correlated with the *Slc6a2* and *Slc6a3* gene expression [50].

Thus, we can take into consideration that changes in expression of the *Slc* DEGs have proven to be valuable and sensible markers in RNA-Seq studies, accurately reflecting specific neuroactivity and metabolic processes in the response to chronic social stress and aggression experiences described in previous publications [13,15,17,20].

Here, we assessed changes in the expression of the *Slc* genes in three specific brain regions (VTA, NAcc, PFC), biochemical processes in which can have a significant impact on the manifestation of behavioral features. When comparing gene expression in the control and aggression experienced male mice, we found a significant decrease in the level of transcription of many *Slc* genes, which is most pronounced in the VTA and NAcc.

We observed the decrease of neurosignaling and metabolic activity in the NAcc and VTA with sets of the *Slc* DEGs. The vast majority of the *Slc* DEGs across three brain regions refer to transmembrane transport. Notably, the genes manifest highly coordinated responses yielding the statistical confidence of non-random expression downturn (Figure 6) in the *Slc* DEGs set of the NAcc, which is a unique projection for such a complex and multifunctional region featuring multiple neurosignaling processes. Paradoxically, the *Slc* network signaling was not so unambiguous for the VTA and PFC regions due to a larger variation in species group expression. Still, the major gene networks incorporating *Slc* DEGs were elucidated there as well.

It is natural to assume that changes in expression profiles of all *Slc* genes in the VTA, NAcc, and PFC regions can be associated with psychosis-like behavioral pathology developed under long-term positive fighting experience, as shown in our works [10,11,14]. Experiments revealed that the number and set of *Slc* DEGs clearly indicate the differences in neurochemical processes in each brain region of the winners. 

It is known that in the VTA dopaminergic neurons play the most significant role in the mechanisms of positive reinforcement of any forms of learned appetitive and motivational behaviors as well as addiction [26,51]. Disturbances in dopaminergic activity are noted in schizophrenia, Parkinson’s disease, attention deficit hyperactivity disorder [52,53], and depression [26,54]. It has been suggested that cellular and molecular adaptations are responsible for a sensitized dopamine activity in this brain region in response to drug abuse [55,56,57]. In the VTA of addicted individuals, the activity of the dopamine-synthesizing enzyme tyrosine hydroxylase increases, and so does the ability of dopaminergic neurons to respond to excitatory inputs [58]. These changes may be accompanied by changes in the expression of addiction-associated dopaminergic genes. Previous experiments in animals also showed that the dopaminergic systems were activated in the VTA of aggressive rats, as dopamine levels were elevated in the NAcc before, during, and after fights [59,60]. Our results described earlier and presented in this work are in good agreement with these concepts. Earlier in our experiments, we showed that the activation of dopaminergic systems in the brain regions of winners [20] is accompanied by increased dopaminergic *Th*, *Slc6a3*, and *Snca* gene expression in the VTA [15,17] and by the development of psychosis-like behavior and addictive symptoms [14,61,62] under positive fighting experience.

This experiment allowed us to expand the list of involved genes, and we can conclude that the upregulation of the genes encoding monoaminergic (***Slc6a2***, ***Slc6a4***) together with the ***Slc6a3*** gene [15,17] and glutamatergic vesicular transporters (***Slc17a7***, ***Slc17a8***) in the VTA may indicate activation of respective neurotransmitter systems in the winners in response to positive fighting experience. We would like to emphasize that the upregulated ***Slc17a7*** gene encodes VGLUT1 protein, which is used as an excitatory synapse marker. Such long-term activation may lead to a deficit of neurotransmitters in the synaptic cleft and be a cause of repetitive aggressive behavior (relapse) in provoking situations. However, downregulation of genes encoding vesicular transporters of acetylcholine (*Slc18a3*), sodium-coupled nucleoside transporters (*Slc28a3*, *Slc29a4*), putative sodium-coupled neutral amino acid transporter (*Slc38a10*), and inhibitory vesicular amino acid GABA and glycine (*Slc32a1*) transporter indicates disturbances of the transport of substrates into synaptic vesicles, thereby exposing them to catabolism processes. We found positive correlation between the expression of genes, which may be due to the activation of glutamatergic neurotransmission, and the inhibition of GABA transport to the vesicles. 

The longest list of *Slc* DEGs was found in the NAcc analysis. All *Slc* DEGs (except ***Slc4a11*** and ***Slc17a7***) were downregulated, suggesting a significant decrease in transport functions for many substrates which are involved in cellular and neurotransmitter processes: glucose and sugar, neurotransmitters, amino acids, inorganic and organic transporters, etc. Our data suggest that the activation of dopaminergic inputs from the VTA, including *Slc* carriers, modulates (decreases) the activity of GABAergic neurons within the NAcc. 

Analysis of cell-specific *Slc* DEGs distribution based on Glia specification patterns (neurons, glial cells (astrocytes, oligodendrocyte precursor, newly formed oligodendrocyte, myelinating oligodendrocyte, microglia), and endothelial cells) from Barres Laboratory data have demonstrated (Table S5) that in the VTA and NAcc most of the *Slc* DEGs may participate in glial processes. In the PFC, a decrease in the level of *Slc* DEGs transcription is associated with processes in glial cells, while an increase in *Slc* DEGs expression is associated with processes in neurons. Overall, the downregulation of the *Slc* DEGs is mainly associated with glial effects influenced by the experience of aggression in mice. Thus, in the PFC, VTA, and NAcc most of the *Slc* DEGs refer predominantly to glial cells (4, 10, and 13 correspondingly; Table S5). While the neural specific DEGs were overall quite a few, in PFC there is a distinct cluster of five neurospecific transporters (Figure 6; Table S5) behaving in a coordinated manner and downregulated in aggressive mice. All of them manifest transmembrane transporter activity. Two are encoding neurotransmitters: (a) proline transporter (*Slc6a7*) involved in GABA neurotransmission cascade and at the same time very distinct in pharmacological specificity, and (b) (*Slc17a8*) glutamate vesicular transporter. As both are downregulated, we may speculate that at least glutamate transmission in the PFC in aggressive species is reduced along with GABA intensity. Notably, two others genes (*Slc26a4* and *Slc5a5*), are involved in iodide transport which is also linked to glutamate/GABA expression alterations in the PFC.

Converging evidence from earlier [59,63,64,65,66,67,68] and more recent [27,69,70] studies have supposed that aggression is rewarding. Positive fighting experience in daily agonistic interactions leads to the activation of dopaminergic systems, in particular in the NAcc, dorsal striatum, and amygdala, as shown earlier [20,59,60]. Our research demonstrates upregulation of the ***Slc6a2***, ***Slc6a3***, ***Slc6a4***, ***Slc17a7***, and ***Slc17a8*** neurotransmitter genes, which may be a consequence of the co-activation of monoaminergic and glutamatergic systems in the VTA, a core region in aggression and addiction circuits. As for inorganic and organic or other carriers, changes in *Slc* gene expression may be a consequence rather than a cause of changes in the activity of neurotransmitters in brain regions.

## 5. Conclusions

Our findings show that in male mice with repeated experience of aggression, altered activity of neurotransmitter systems, especially the dopaminergic one, leads to a restructuring of metabolic and neurotransmitter processes in a way specific for each brain region. It can be assumed that an overall decrease in the expression of *Slc* genes may be ascribed to the chronic stimulation of the reward systems [14,61].

When analyzing these findings, it was natural to ask: what processes lead to the downregulation of most *Slc* genes encoding different types of substrate-specific transporter proteins in the VTA and NAcc? It is very important to glean knowledge about this phenomenon because transporters are considered to be promising therapeutic targets in the treatment of many diseases [5]. It has been shown that natural rewards, such as food (glucose) and sex, as well as pharmacological manipulations are accompanied by the activation of dopaminergic systems and reduce the electrophysiological excitability of GABA-containing MSNs in the NAcc [71,72]. In our model, aggression is developed against the background of positive reinforcement, and a decrease in the expression of the *Slc* genes may indicate the inactivation of the NAcc, which is consistent with literature data that rewarding stimuli reduce the activity of NAcc MSNs, whereas aversive stimuli increase the activity of these neurons through inhibitory functions of the NAcc D2 receptor, which plays a critical role in the reward mechanism [71]. If so, then the key genes may rather be the genes that are responsible for electrical impulse conduction, i.e., genes encoding inorganic substrate transporters. These data should be taken into consideration to explain the overall decrease in the expression of most *Slc* genes in the VTA and NAcc.

Thus, the psychosis-like state developed under repeated positive fighting experience and accompanied by addiction-like symptoms shown in our winners [10,14,61], similar to chronic drug use [39,73], involves alterations in gene expression in the mesocorticolimbic systems. Dopamine-dependent rewarding attenuates the overall excitability of GABAergic neurons, and these processes are exhibited in the decreased expression of many Slc* genes encoding transporters of amino acids, glucose, nucleoside sugars, neurotransmitters, vesicular transporters, and inorganic and organic ions, which leads to changes in signaling cascades through reduced activation of GABAergic MSNs in the NAcc, and thereby to decreased inhibitory control of aggression [71].

We should stress that the *Slc* gene family vector space manifests resolution high enough to accurately separate both brain regions and contrast groups in virtually all gene regions, which renders its application as a highly specific marker system outlined in this and other publications. Although the interpretation of these findings warrants further investigation with the inclusion of other correlated / relevant genes, altered expression of Slc genes set may be treated as primary marker scaffold in brain RNA-Seq studies. 

However, according to available data, no contemporary drugs have been developed specifically to activate SLC transporters. It is very important to find new targets for correction of metabolic and neurotransmitter processes implicated in the pathological states. Altered expression of the *Slc* genes characterized by the largest number of correlations with other gene expression may serve as a prognostic target and a tool to search for new drug generation.

The Innovative Medicines Initiative Consortium RESOLUTE [74] has begun developing tools and producing datasets to develop new approaches to studying the SLC superfamily as a possible drug target class. We very much hope that the results of our study will be a useful contribution to this research.

## 6. Limitations

One of the limitations of whole transcriptome analysis is the high cost of research, which does not allow for the taking of a large number of samples, in our case, in comparative analysis of transcriptomes of control and experimental samples for statistical analysis. Nevertheless, a number of studies have demonstrated that the results of the RNA-Seq analysis show high levels of reproducibility for both technical and biological replicas [75,76]. Comparison of the RNA-Seq results with the qPCR data show a high correlation (r = 0.98), which was obtained by various authors, including in our previous studies [75,77]. Therefore, the small number of repetitions that are commonly used in this analysis is usually not considered a critical limitation of this method. 

Additionally, we would like to point out the fact, as second limitation, that genes that are only slightly outside the accepted threshold of a statistically significant value of *q* < 5% fall out of the field of view of researchers. However, we believe that even small changes in the level of gene expression can have physiologically important consequences. This is indicated by the fact that cellular signaling can be dose-dependent [78]. Considering that a small number of samples were used in our work in order not to lose functionally important genes, which for random reasons were not included in the list of differentially expressed genes in accordance with the *q* criterion, we present data in the article taking into account not only the q value, but also the *p* value.

## Figures and Tables

**Table 1:** *Slc* DEGs encoding the transporters in different brain regions according to type of substrate category for every gene.

Substrate Category *	The *Slc* Transporters, Cotransporters, or Symporter Genes		
	VTA	NAcc	PFC
Amino acids: glycine, proline, and glutamate, etc.	↓*Slc38a10*	↓*Slc1a5,* ↓*Slc7a11,* ↓*Slc38a2,*↓*Slc38a11*	**↑*Slc38a2***
Glucose, nucleotide sugars	↓*Slc5a11,* ↓*Slc35e1*	↓*Slc2a4,* ↓*Slc47a1*	↓*Slc35f4*
Metals: zinc, magnesium, copper	↓*Slc39a13*	↓*Slc31a1,* ↓*Slc41a1*	**↑*Slc39a2***
Neurotransmitters: noradrenaline, serotonin, glutamate, choline, GABA, proline	↑*Slc6a2,*↑Slc6a3 **, ↑*Slc6a4*	↓*Slc5a7,* ↓*Slc6a12,* ↓*Slc6a13,* ↓*Slc6a20a*	↓*Slc6a7*
Vesicular transporter of neurotransmitter glutamate, acetylcholine, GABA, glycine and amino acid	**↑ *Slc17a7,* ↑*Slc17a8****,* ↓*Slc18a3,* ↓*Slc32a1,*	**↑ *Slc17a7***	↓*Slc17a8*
Inorganic ions: chloride, bicarbonate, hydroxide, sulfate, potassium, sodium, phosphate, monocarboxylate jodide, etc.	↓*Slc12a4,* ↓*Slc12a9*	↓*Slc4a2*, ↓*Slc4a5*, **↑*Slc4a11,*** ↓*Slc5a5, *↓*Slc12a7,* ↓*Slc13a3*, ↓*Slc13a4*, ↓*Slc16a12*	↓*Slc5a5**,***↑*Slc16a12,*↑*Slc16a13****,* ↓*Slc26a4*
Organic anions and cations, oligopeptide	**↑*Slc22a3****,* ↓*Slco3a1*	↓*Slc22a2,* ↓*Slc22a6,* ↓*Slc22a8,* ↓*Slco5a1*	
Nucleosides	↓*Slc28a3,*↓*Slc29a4*	↓*Slc29a4*	**↑*Slc29a3***
Mitochondria		↓*Slc25a18*	**↑*Slc25a47***
Fatty acids	↓*Slc27a1*		

Emphasized line: common genes for regions; **Bold** font: **↑**upregulation; Regular font: **↓**downregulation; * Substrate category for every gene was taken from [1,2,3], ** [17,19].

## Data Availability

The additional statistics of data obtained used to support the findings of this study are available from Supplementary Table S1: differentially expressed *Slc* genes in FPKM units and are cited at relevant places within the text. The other datasets generated during the current study are available from the corresponding author on reasonable request.

## Supplementary Materials

The following are available online at https://www.mdpi.com/article/10.3390/genes12071099/s1: Table S1: Expression level (FPKM) of the *Slc* DEGs in the VTA, Nacc, and PFC; Table S2: AHС based on expression profiles of the *Slc* DEGs in the VTA, NAcc, and PFC; Table S3: Correlation coefficients between the *Slc* DEGs in the VTA, NAcc, and PFC; Table S4: Agglomerative hierarchical clustering (AHC) genes encoding *Slc* transporters and neurotransmitter receptors in the NAcc in winners and control mice; Table S5: Cell-specific DEGs distribution based on Glia specification from (Zhang et al., 2014) data.

## Abbreviations

MSN	medium spiny neurons
*Slc*	solute carrier family
PCA	principal component analysis
AHC	agglomerative hierarchical clustering
FPKM	fragments per kilobase of transcript per million mapped reads
VTA	ventral tegmental area
NAcc	nucleus accumbens
PFC	prefrontal cortex
W	winners
C	controls
